# Worldwide antipsychotic drug search intensities: pharmacoepidemological estimations based on Google Trends data

**DOI:** 10.1038/s41598-021-92204-0

**Published:** 2021-06-23

**Authors:** Richard Ågren

**Affiliations:** Department of Neuroscience, Karolinska Institutet, 171 77 Stockholm, Sweden

**Keywords:** Epidemiology, Health care economics

## Abstract

Prescription patterns of antipsychotic drugs (APDs) are typically sourced from country-specific data. In this study, a digital pharmacoepidemiological approach was used to investigate APD preferences globally. Publicly available data on worldwide web search intensities in Google for 19 typical and 22 atypical APDs were temporally and spatially normalized and correlated with reported prescription data. The results demonstrated an increasing global preference for atypical over typical APDs since 2007, with quetiapine, olanzapine, risperidone, and aripiprazole showing the largest search intensities in 2020. Cross-sectional analysis of 122 countries in 2020 showed pronounced differences in atypical/typical APD preferences that correlated with gross domestic product per capita. In conclusion, the investigation provides temporal and spatial assessments of global APD preferences and shows a trend towards atypical APDs, although with a relative preference for typical APDs in low-income countries. Similar data-sourcing methodologies allow for prospective studies of other prescription drugs.

## Introduction

Schizophrenia and bipolar disorder inflict excruciating global disease burdens^1,2^. Antipsychotic drugs (APDs) are the mainstay of treatment and reduce the overall mortality in schizophrenia^3^. APDs are categorized as typical and atypical, with drugs in both groups sharing varying degrees of dopamine D2 receptor occupancy^4^. Additionally, APDs demonstrate multitarget occupancies, including, but not limited to, serotonergic, muscarinic, histaminergic, and adrenergic receptors^5^. Typical APDs are associated with extrapyramidal side effects (EPS), sedation, and anticholinergic responses. Clozapine-based atypical APDs can retain these side effects to varying degrees, in addition to metabolic and endocrine side effects^6,7^.

Globally, numerous medical and non-medical factors are likely to affect the selection of typical or atypical APDs for individual patients. Interestingly, early cost-effectiveness analyses have failed to demonstrate the superiority of atypical over typical APDs^8–10^. However, with regard to clinical efficacy, the four atypical APDs amisulpride, clozapine, olanzapine, and risperidone have been proposed to be superior to typical APDs^11^. Moreover, atypical APDs have been associated with lower risks of tardive dyskinesia^12^, decreased mortality^13^, and increased self-reported quality of life^14^. On a network meta-analysis level, differences in APD side effects were more pronounced than differences in efficacy^6^.

Variations in international APD prescription patterns are evident; for example, increasing prescription of atypical APDs has been shown in a study including 16 countries^15^. In addition, clozapine prescription has been shown to differ between 17 countries, with a possible underuse in some countries^16^. Studies from the Research on Asian Psychotropic Prescription Patterns (REAP) consortium support differences in clozapine and general APD prescriptions in a number of Asian countries^17,18^. Despite collaborative studies, the global prescription patterns of atypical and typical APDs remain unclear.

Previously, internet search patterns have been utilized to evaluate seasonal epidemics such as influenza and chicken pox^19,20^. Similar approaches have also been used to investigate drug prescription patterns with temporal and spatial resolutions^21^. In the current study, the relative search intensities for 19 typical and 22 atypical APDs in years 2004-2020 were retrieved using Google Trends. The hypothesis was that the interests of patients, healthcare professionals, researchers, and public for different APDs provided indirect temporal and spatial information about the preferences for and use of specific APDs. In addition, the worldwide cross-sectional APD search intensities per country were analyzed for 2020, revealing spatial differences in atypical APD preferences. Finally, to evaluate the influence of non-medical aspects on APD preferences, atypical versus typical APD search intensities were correlated with gross domestic product (GDP) on a country-specific basis.

## Results

### Search intensity in relation to antipsychotic drug approval and prescription

**Figure 1:**
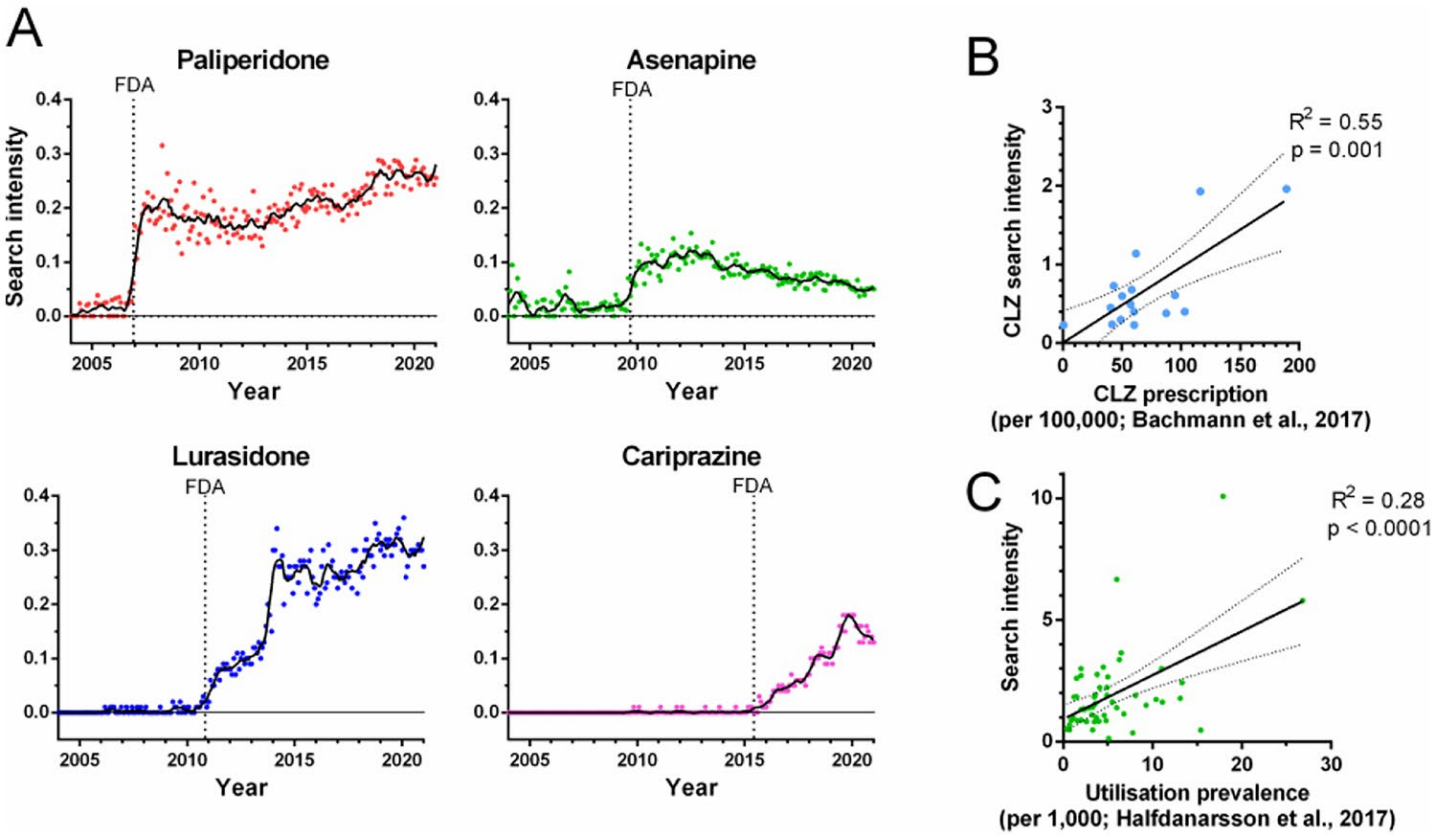
Evaluation of antipsychotic drug (APD) search intensities in relation to approval and country-wise prescription. Search intensities are normalized to the maximal intensity of haloperidol, in 2004–2020. Data from “low search intensity regions” are included. (**A**) Worldwide search intensities for recently approved APDs (FDA approval date; dotted line) are paliperidone (Dec 2006), asenapine (Aug 2009), lurasidone (Oct 2010), and cariprazine (May 2015). Smooth polynomials of order 2 were adapted to 6 adjacent points (black traces). (**B**) Clozapine search intensities in relation to clozapine prescription for 16 countries in 2014, as reported by Bachmann *et al.*^16^ (linear regression, R^2^ = 0.55 and *p* = 0.001). For USA, the reported public and private insurance groups were averaged^16^. Clozapine search intensities were not available for Iceland. (**C**) APD search intensities in relation to prescription of the five most common APDs in 13 countries in year 2014^15,16^ (linear regression, R^2^ = 0.28 and *p* < 0.0001). Levomepromazine and prochlorperazine were not included because of the exclusion of these drugs. Chlorpromazine was excluded for Japan because of a disproportionate search intensity. Search intensities were not available for Iceland. For USA, the public insurance group was used^15^. Dotted lines represent 95% CI. CLZ, clozapine. See Supplementary Tables S1, S2, and S3 for underlying search intensities and respective publications for prescription data^15,16^.

Google Trends-derived search intensities in 2004-2020 for 19 typical and 22 atypical APDs were normalized to the maximum of haloperidol. Initially, the temporal overlap between search intensities and Food and Drug Administration (FDA) approval was evaluated. For the atypical APDs paliperidone, asenapine, lurasidone, and cariprazine, search intensities increased beginning around the time point of FDA approval (Fig. 1A; Supplementary Table S1). Three additional FDA approvals showed similar patterns (Supplementary Fig. S1A), although the evolution of search intensities varied substantially between the APDs (Supplementary Fig. S1B).

The spatial accuracy of the APD search intensities was investigated in relation to the prescription data. Clozapine prescriptions in 17 countries were reported by Bachmann *et al.*, who showed a 315-fold larger use per 100,000 individuals in Finland than in Japan^16^. Normalized search intensities for clozapine in 2014 for the included countries correlated with the corresponding prescriptions in the same year^16^ (Fig. 1B; Supplementary Table S2). Similarly, a correlation was observed when data sourcing from “low search intensity regions” was excluded (Supplementary Fig. S2; Supplementary Table S2). Moreover, search intensities were compared to APD prescription data from 13 countries^15^. Prescription frequencies for the five most prescribed APDs correlated with the normalized search intensities (Fig. 1C; Supplementary Table S3). In conclusion, normalized APD search intensities were shown to temporally and spatially reflect prescriptions.

### Global antipsychotic drug trends in 2004-2020

**Figure 2:**
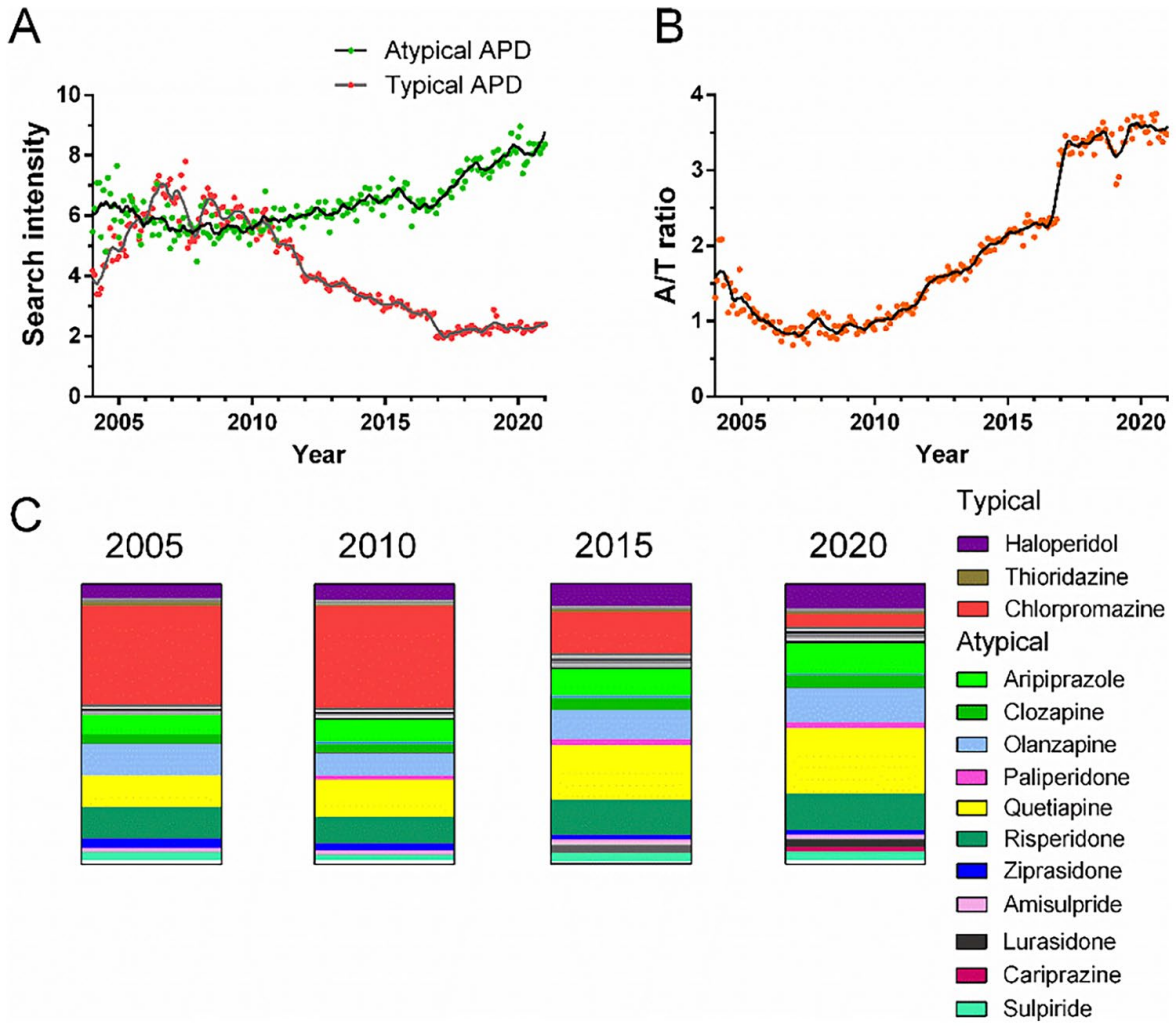
Global antipsychotic drug (APD) search intensities in 2004-2020. Data were normalized to the maximum search intensity of haloperidol. (**A**) Global typical (n = 19) and atypical APD (n = 22, see “Methods” section) search intensities over time. (**B**) Global atypical/typical APD search intensity (A/T) ratio over time. (**C**) Vertical slices demonstrate the relative search intensities for typical and atypical APDs in 2005, 2010, 2015, and 2020, with the most common APDs specified. See Supplementary Figs. S5–S6 and Supplementary Table S1 for search intensities of all included typical and atypical APDs.

The search intensities of atypical APDs increased gradually over time, whereas those of typical APDs decreased from approximately 2006 (Fig. 2A and Supplementary Fig. S3). Accordingly, the atypical/typical APD search intensity (A/T) ratio increased from approximately 2007 (Fig. 2B) based on search intensities normalized to haloperidol. Corresponding search intensity normalizations to the atypical APD risperidone showed a similar A/T ratio over time (Supplementary Fig. S4A; Supplementary Table S4), which correlated with haloperidol-based normalization (Supplementary Fig. S4B).

The search intensity of the most common typical APD, chlorpromazine, decreased, whereas that of the atypical APDs quetiapine and aripiprazole increased (Fig. 2C). Notably, only a fraction of all APDs displayed search interests above 10% of the reference haloperidol (Supplementary Figs. S5 and S6), suggesting that a small number of APDs represented the majority of all prescriptions. The partial dopamine D2 receptor agonists, which could be considered separate APD entities^22^, showed an increasing trend over time (Supplementary Fig. S7).

### Country-wise atypical antipsychotic drug search intensities in 2020

**Figure 3:**
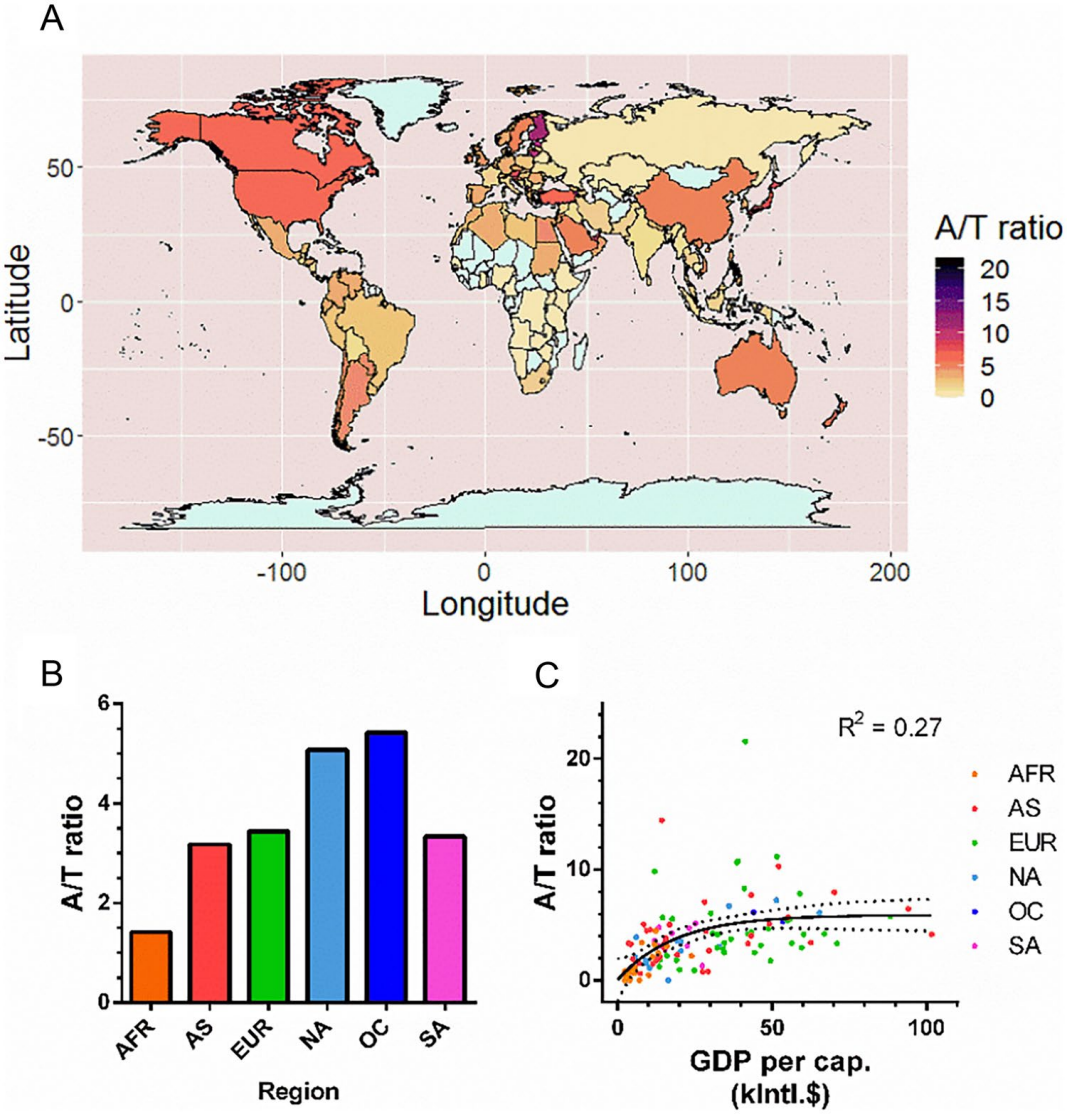
Worldwide atypical/typical antipsychotic drug (APD) search intensity (A/T) ratios and relation to gross domestic product (GDP). Data from 2020. In total, 122 countries with complete data were included. (**A**) Choropleth of A/T ratios per country. Light gray, data not available. (**B**) Comparison of population-weighted A/T ratios per continent. (**C**) A/T ratio as a function of GDP per capita (purchasing power parity). A monoexponential function was fitted to all data points (black; R^2^ = 0.27), with 95% C.I. (dotted). AFR, Africa (n = 21); AS, Asia (n = 37); EUR, Europe (n = 37); NA, North America (n = 14); OC, Oceania (n = 2); SA, South America (n = 11). See Supplementary Table S5 for search intensity data. The choropleth graph was rendered using R, version 4.0.4, and the toolbox ggplot2^23^.

Based on the correlation between internet search intensities and international clozapine (Fig. 1B) or APD prescription (Fig. 1C), country-specific atypical and typical APD search intensities for 2020 were normalized to haloperidol. The global A/T ratios demonstrated pronounced differences between and within the regions (Fig. 3A; Supplementary Table S5). Geographical analyses, adjusted for population size, demonstrated falling A/T ratios from Oceania > North America > Europe > South America > Asia > Africa (Fig. 3B). Similar trends were observed if low-intensity search regions were excluded (Supplementary Fig. S8A; Supplementary Table S6), consistent with the findings in Fig. 3B (and Supplementary Fig. S8B). To evaluate the relationship with socioeconomic determinants, the A/T ratios of 122 countries were compared to GDP per capita (purchasing power parity; International Monetary Fund, 2018), to which a monoexponential function was fitted (Fig. 3C). In addition, the linear regression between A/T ratios and GDP showed a significant correlation (Supplementary Fig. S9). Individual analyses of A/T ratios and GDP per region demonstrated monoexponential relationships for North America, Asia, and Africa (Supplementary Fig. S10). In conclusion, atypical/typical APD search intensity ratios, reflecting APD preferences, differ between countries and regions and are associated with GDP per capita.

## Discussion

Information regarding the global use of APDs remains largely unelucidated. Based on a digital epidemiology approach, using publicly accessible temporal and spatial internet search query data, three main conclusions were drawn. First, the search intensities of APDs correlated with FDA approval time points and prescription data, lending support to the methodological approach. Second, atypical APD preferences increased worldwide in the last 15 years, related to both an increase in atypical and a decrease in typical APD search intensities. Finally, in 2020, the search intensities for atypical and typical APDs differed widely between countries and regions. Increased atypical APD preferences were associated with higher GDP, indicating that socioeconomic factors may associate with APD prescription.

Previous global investigations of APD prescription have suggested an increase in the use of atypical APDs^15^. The REAP studies show a similar trend in Asian countries, with a transition from 67.8 to 31.3% of first-generation APD prescriptions from 2001 to 2016^24,25^. In line with the reported trend, the A/T ratio increased from approximately 2006 onwards. The APD search intensities in 2004-2020 indicate a preference for two typical drugs, chlorpromazine and haloperidol. In line with the World Health Organization’s (WHO) list of essential medicines, the typical APDs haloperidol, chlorpromazine, and fluphenazine are deemed essential^26^. Fluphenazine is listed as a long-acting injectable, which, considering the method of administration, may explain the lower search intensity.

For atypical APDs, distinct search intensities for quetiapine, risperidone, olanzapine, aripiprazole, and clozapine were identified in 2004-2020. The dominant quetiapine search intensity from 2008 onwards (Fig. 2C, Supplementary Fig. S6) may be related to the development of both rapid- and slow-release drug formulations, and the wide range of clinical indications, including psychosis, bipolar disorder, and as an adjunct to treatment-resistant major depressive disorder^27^. Similarly, numerous other APDs are indicated for several psychiatric disorders and may be prescribed off-label^28–30^. For the included 11 typical and 14 atypical FDA-approved APDs, the average number of indications (calculated for schizophrenia, bipolar disorder, autism spectrum disorder/tics, major depressive disorder, nausea, and other indications) were 1.7 and 2.0 respectively. Although this suggests a slight bias in favor of atypical APDs, this is unlikely to explain the differences observed in the A/T ratio alone (Fig. 2B).

The proposed benefits of atypical APDs such as decreased risk of tardive dyskinesia, decreased mortality, and increased quality of life^12–14^ are balanced by the higher cost and lower availability, in line with previous findings of preferred prescription of atypical APDs^15^. Individual investigations of individual mental health centers lend support to the unavailability of atypical APDs in Africa^31^ and the importance of atypical APDs in the pharmacological armamentarium is underscored by the inclusion of risperidone and clozapine in the WHO list of essential medicines^26^. The observed relationship between GDP per capita and the ratio between atypical and typical APD search intensities (Fig. 3C and Supplementary Fig. S9) may reflect how higher costs or unavailability of atypical APDs affect usage. However, external factors such as pharmaceutical marketing may confound this relationship and influence the results. Further complexity is added by various regulations; for example, “direct-to-consumer” drug marketing is largely prohibited outside the United States^32^. In addition, GDP per capita and A/T ratios showed no clear relation for countries in Europe or South America, which for the former may be related to, for example, centralized drug approval within the European Union^33^.

For APDs approved by the FDA after 2004, data showed differential evolution of search intensities (Fig. 1A, Supplementary Fig. S1A and B). Cariprazine and lurasidone, FDA-approved in 2010 and 2015, respectively, passed the peak search intensity of asenapine, which was approved in 2009. In light of previous studies showing a relationship between financial search intensities (e.g., cryptocurrency^34^) and pricing, search trends for recently approved drugs may potentially contribute to financial forecasting of the corresponding drug revenues.

The search intensities associated with the international prescription of clozapine and other APDs^15,16^. However, the analyzed search intensities may not fully reflect true prescription rates or use, but rather demonstrate the underlying preference or interest for each drug. Differences between search intensities and prescriptions may arise from various sources, including the absence of search interest (e.g., restricted access or awareness of Google resources) or absence of representative prescription data (e.g., discrepancies between public and private health care systems). Additionally, Google Trends does not disclose details regarding absolute search intensities, underlying calculations, or thresholds for inclusion. Therefore, the data were normalized using a reference search index (see “Methods” section).

The access and use of Google search engines vary globally, which may lead to low search volumes or lack of data from select regions. However, the use of country-specific atypical/typical APD search intensity ratios provides relative preferences. Finally, the present investigation included 19 typical and 22 atypical APDs, selected to cover the majority of the most prevalent dopamine D2 receptor-targeting APDs used for psychiatric disorders, as reported in the literature^15^. Nevertheless, the selection does by far represent all globally used APDs in 2004–2020, and the atypicality of a number of APDs remains disputed.

A global pharmacoepidemiological investigation of APDs was performed based on publicly available Google search data. The results suggest that the worldwide popularity of atypical APDs has increased since 2007. Despite this, the popularity of atypical versus typical APDs varies between geographical regions, with a positive relationship between gross domestic product and preference for atypical APDs. The conclusions strengthen the view of worldwide differences in APD prescription patterns.

## Methods

### Google Trends data sourcing

The publicly available service Google Trends provides relative temporal and spatial scores of up to five Google search indices (www.google.com/trends, accessed: 2020-02-01). The data sampling frequency is dependent on the time range; from January 2004, monthly data are available. Breakdown by search region provides an option of incorporating “low search volume regions”, which has been compared with the exclusion of these regions. The relative measurements of search intensities, provided as scores 0-100, as well as cut-off values for intensities or high and low search volumes regions, remain undefined.

For analysis, search groups of 19 typical (haloperidol, perphenazine, pimozide, thiothixene, thioridazine, chlorpromazine, droperidol, fluphenazine, loxapine, trifluoperazine, periciazine, pipotiazine, timiperone, flupentixol, zuclopenthixol, thioproperazine, cyamemazine, pipamperone, and chlorprothixene) and 22 atypical APDs (aripiprazole, asenapine, clozapine, iloperidone, olanzapine, paliperidone, quetiapine, risperidone, ziprasidone, zotepine, sertindole, blonanserin, amisulpride, nemonapride, sultopride, perospirone, melperone, lurasidone, cariprazine, brexpiprazole, sulpiride, and lumateperone) were included. The selection intended to cover the majority of the globally most prevalent dopamine D2 receptor-targeting APDs used for psychiatric disorders, as reported in the literature by Hálfdánarson *et al.*^15^. All search terms were of the subcategory “drug” or “substance”, referring to a specific substance in several languages. Temporal data for the “worldwide” region were retrieved from January 2004 to January 2021, with monthly sampling frequency. Spatial data were retrieved for the full years 2014 and 2020 for regular and “low search volume” regions. The search intensities displayed as < 1% were reduced to 0.

### Normalization of search intensities

To allow for temporal comparison of search intensities, the common term “haloperidol” was included in all data retrievals. Alternatively, “risperidone” was used for comparison (see Supplementary Fig. S4). Four additional drugs were included, and the search intensities were normalized to the maximum of “haloperidol” (see Supplementary Fig. S11A). For spatial data, search intensities were retrieved for the common term “haloperidol” and four additional APDs, for all countries, at a given year (2014 for Fig. 1B and C, and 2020 for Fig. 3), and then normalized to “haloperidol” for each country (see Supplementary Fig. S11B).

### Data analysis

Ratios between the normalized search intensities for all typical (n = 19) and atypical APDs (n = 22) were calculated, and linear or monoexponential curve fitting was performed using GraphPad 6 (Prism software). Correlations were analyzed using the Pearson’s correlation coefficient. *p* < 0.05 was considered statistically significant.

## Supplementary Information

Supplementary Figures.

Supplementary Table S1.

Supplementary Table S2.

Supplementary Table S3.

Supplementary Table S4.

Supplementary Table S5.

Supplementary Table S6.

## Data Availability

All data generated or analysed during this study are included in this published article (and its Supplementary Information files).

## References

[1] Ferrari AJ (2016). The prevalence and burden of bipolar disorder: findings from the global burden of disease study 2013. Bipolar Disord..

[2] Charlson FJ (2018). Global epidemiology and burden of schizophrenia: findings from the global burden of disease study 2016. Schizophr. Bull..

[3] Torniainen M (2015). Antipsychotic treatment and mortality in schizophrenia. Schizophr. Bull..

[4] Nord M, Farde L (2011). Antipsychotic occupancy of dopamine receptors in schizophrenia. CNS Neurosci. Ther..

[5] Roth BL, Sheffler DJ, Kroeze WK (2004). Magic shotguns versus magic bullets: selectively non-selective drugs for mood disorders and schizophrenia. Nat. Rev. Drug Discov..

[6] Huhn M (2019). Comparative efficacy and tolerability of 32 oral antipsychotics for the acute treatment of adults with multi-episode schizophrenia: a systematic review and network meta-analysis. Lancet.

[7] Nasrallah HA (2008). Atypical antipsychotic-induced metabolic side effects: insights from receptor-binding profiles. Mol. Psychiatry.

[8] Davies LM (2007). Cost-effectiveness of first- v. second-generation antipsychotic drugs: results from a randomised controlled trial in schizophrenia responding poorly to previous therapy. Br. J. Psychiatry.

[9] Rosenheck RA (2006). Cost-effectiveness of second-generation antipsychotics and perphenazine in a randomized trial of treatment for chronic schizophrenia. Am. J. Psychiatry.

[10] Hanrahan P, Luchins DJ, Fabian R, Tolley G (2006). Cost-effectiveness of atypical antipsychotic medications versus conventional medication. Expert Opin. Pharmacother..

[11] Leucht S (2013). Comparative efficacy and tolerability of 15 antipsychotic drugs in schizophrenia: a multiple-treatments meta-analysis. Lancet.

[12] Carbon M, Kane JM, Leucht S, Correll CU (2018). Tardive dyskinesia risk with first- and second-generation antipsychotics in comparative randomized controlled trials: a meta-analysis. World Psychiatry.

[13] Zagozdzon P, Goyke B, Wrotkowska M (2016). Mortality rates in users of typical and atypical antipsychotics: a database study in Poland. Drugs Real World Outcomes.

[14] Grunder G (2016). Effects of first-generation antipsychotics versus second-generation antipsychotics on quality of life in schizophrenia: a double-blind, randomised study. Lancet Psychiatry.

[15] Halfdanarson O (2017). International trends in antipsychotic use: a study in 16 countries, 2005–2014. Eur. Neuropsychopharmacol..

[16] Bachmann CJ (2017). International trends in clozapine use: a study in 17 countries. Acta Psychiatr. Scand..

[17] Chong M-Y (2004). Antipsychotic drug prescription for schizophrenia in East Asia: rationale for change. Psychiatry Clin. Neurosci..

[18] Xu S-W (2020). Clozapine prescription pattern in patients with schizophrenia in Asia: The REAP survey (2016). Psychiatry Res..

[19] Yang S, Santillana M, Kou SC (2015). Accurate estimation of influenza epidemics using Google search data via ARGO. Proc. Natl. Acad. Sci..

[20] Bakker KM, Martinez-Bakker ME, Helm B, Stevenson TJ (2016). Digital epidemiology reveals global childhood disease seasonality and the effects of immunization. Proc. Natl. Acad. Sci..

[21] Lippi G, Mattiuzzi C, Cervellin G, Favaloro EJ (2017). Direct oral anticoagulants: analysis of worldwide use and popularity using Google Trends. Ann. Transl. Med..

[22] Lieberman JA (2004). Dopamine partial agonists: a new class of antipsychotic. CNS Drugs.

[23] Wickham H (2011). ggplot2. Wiley Interdiscip. Rev. Comput. Stat..

[24] Xiang YT, Ungvari GS, Correll CU, Chiu HFK, Shinfuku N (2016). Trends in the access to and the use of antipsychotic medications and psychotropic co-treatments in Asian patients with schizophrenia. Epidemiol. Psychiatr. Sci..

[25] Dong M (2019). Prescription of antipsychotic and concomitant medications for adult Asian schizophrenia patients: findings of the 2016 research on Asian psychotropic prescription patterns (REAP) survey. Asian J. Psychiatry.

[26] World Health O (2017). WHO Model List of Essential Medicines, 20th List (March 2017, Amended August 2017).

[27] Al-Jurdi RK, Dixit LA, Sajatovic M (2010). Role of extended release quetiapine in the management of bipolar disorders. Neuropsychiatr. Dis. Treat..

[28] Glick ID, Murray SR, Vasudevan P, Marder SR, Hu RJ (2001). Treatment with atypical antipsychotics: new indications and new populations. J. Psychiatr. Res..

[29] O’Brien PL, Cummings N, Mark TL (2017). Off-label prescribing of psychotropic medication, 2005–2013: an examination of potential influences. Psychiatr. Serv..

[30] Carton L (2015). Off-label prescribing of antipsychotics in adults, children and elderly individuals: a systematic review of recent prescription trends. Curr. Pharm. Des..

[31] Wagenaar BH (2015). The availability of essential medicines for mental healthcare in Sofala, Mozambique. Glob. Health Action.

[32] Ventola CL (2011). Direct-to-consumer pharmaceutical advertising: Therapeutic or toxic?. P T Peer Rev J Formul. Manag..

[33] Van Norman GA (2016). Drugs and devices: comparison of European and U.S. approval processes. JACC Basic Transl. Sci..

[34] Kristoufek L (2013). Can Google Trends search queries contribute to risk diversification?. Sci. Rep..

