# Supplementary material for: Worldwide antipsychotic drug search intensities: pharmacoepidemological estimations based on Google Trends data
Source: Sci Rep. 2021 Jun 23;11:13136. doi: 10.1038/s41598-021-92204-0 (PMC8222314; doi:10.1038/s41598-021-92204-0)
Supplement: Supplementary file 1 — Supplementary Figures. [file 41598_2021_92204_MOESM1_ESM.pdf]

Supplementary information

Worldwide antipsychotic drug search intensities: pharmacoepidemological estimations based on Google Trends data

Richard Ågren[1]

[1]Department of Neuroscience, Karolinska Institutet, 171 77, Stockholm, Sweden.
Email: richard.agren@ki.se

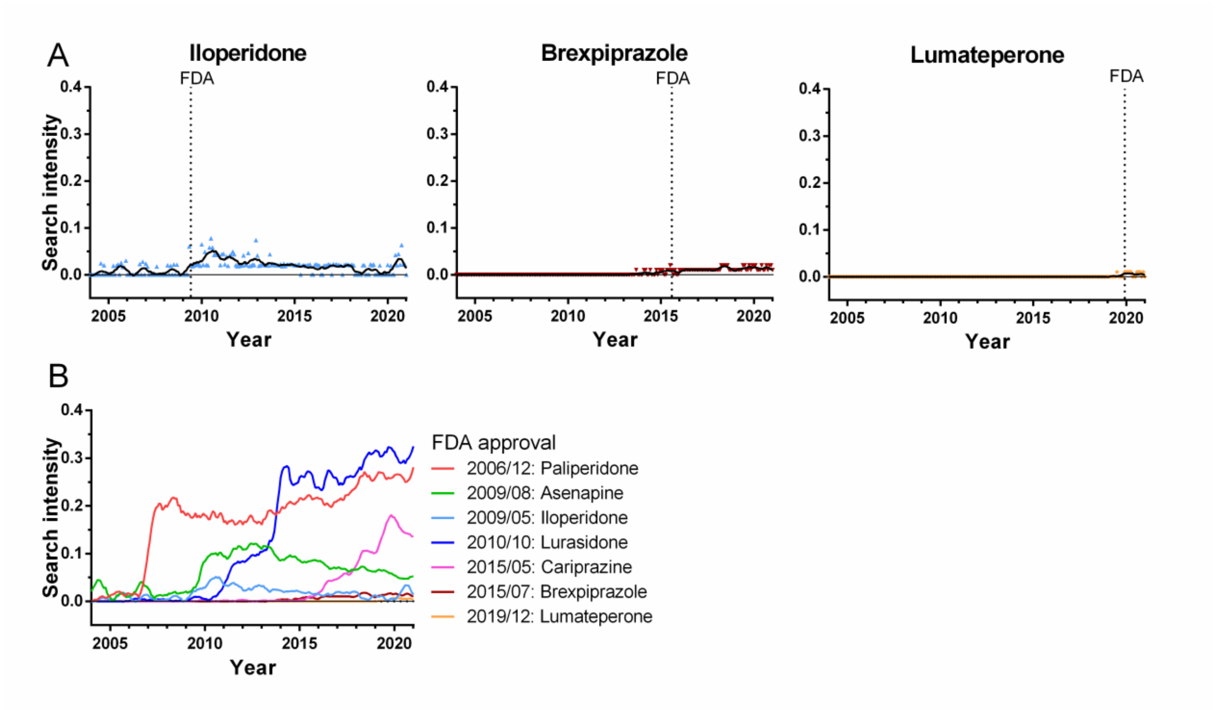

Supplementary Figure S1. Search intensities of recently approved APDs in 2004-2020. A) Iloperidone, brexpiprazole, and lumateperone, normalized to the max search intensity of haloperidol (in 2004-2020). Smooth polynomials of order 2 were adapted to 6 adjacent points (black traces). B) Smooth polynomials and FDA approval dates of all included APDs approved after Jan 2004 show diverse search intensity evolutions over time.

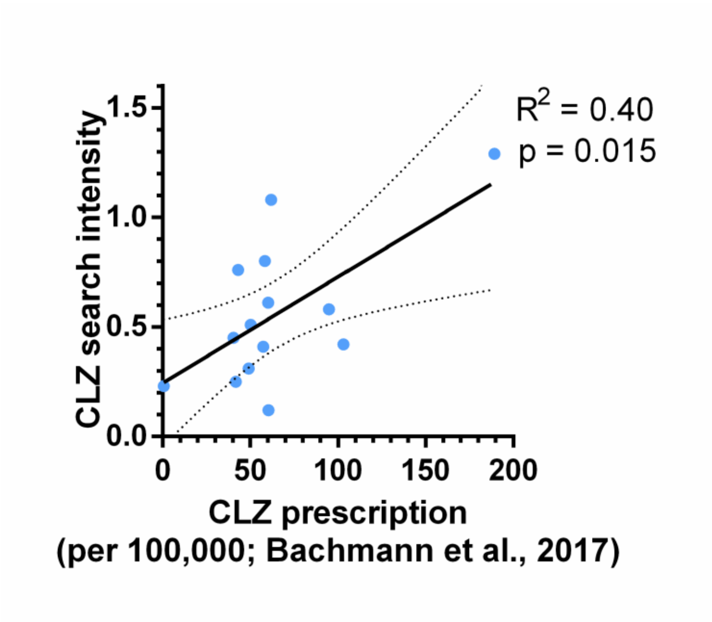

Supplementary Figure S2. Clozapine search intensity as a function of prescription, with "low search intensity regions" excluded. Clozapine search intensities in 2014 are expressed as haloperidol equivalents. Prescription data from 14 countries were retreived from Bachmann *et al.* [1] Iceland, New Zealand, and Taiwan were excluded due to absence of search intensity data. US prescription data from the public and private sectors were averaged. A linear regression with 95% C.I. (dotted) was fitted to the data (R^2 = 0.40 and p = 0.015).

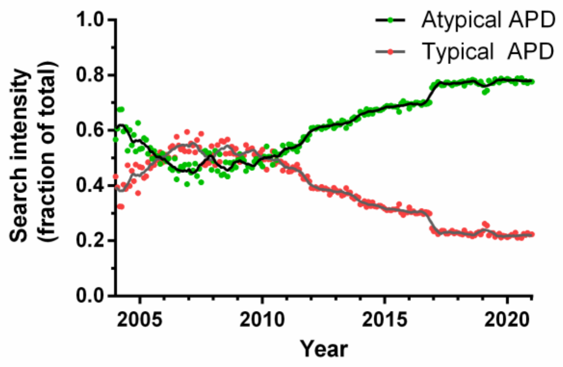

Supplementary Figure S3. Fraction of atypical and typical APD search intensities in 2004-2020. Search intensities were initially normalized to haloperidol. The data represent worldwide search intensities for all atypical (n = 22) and typical APDs (n = 19) as a fraction of all APDs (n = 41). Smooth polynomials of order 2 were adapted to 6 adjacent points (black, atypical; gray, typical).

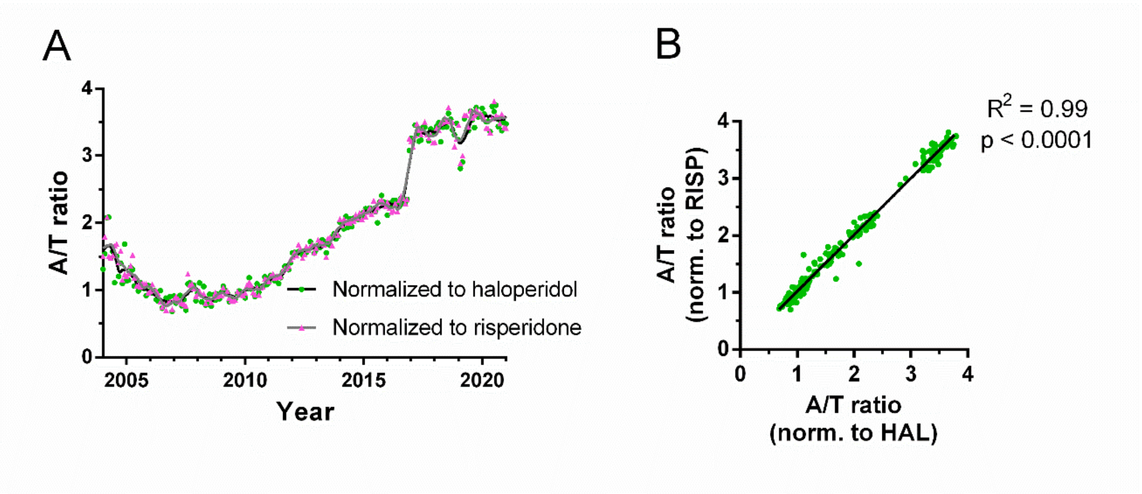

Supplementary Figure S4. Role of normalization search terms for temporal analysis. The atypical and typical APD search intensities (A/T) ratio was calculated based on individual drugs (n = 41) normalized to risperidone (RISP), in addition to haloperidol (HAL). A) Temporal relationship between A/T ratios, normalized to RISP and HAL. Smooth polynomials of order 2 were adapted to 6 adjacent points (black, atypical; gray, typical). B) Correlation between temporal relationship of A/T ratios, normalized to RISP and HAL. A linear regression was fitted to the data ($R^2 = 0.99$ and $p < 0.0001$).

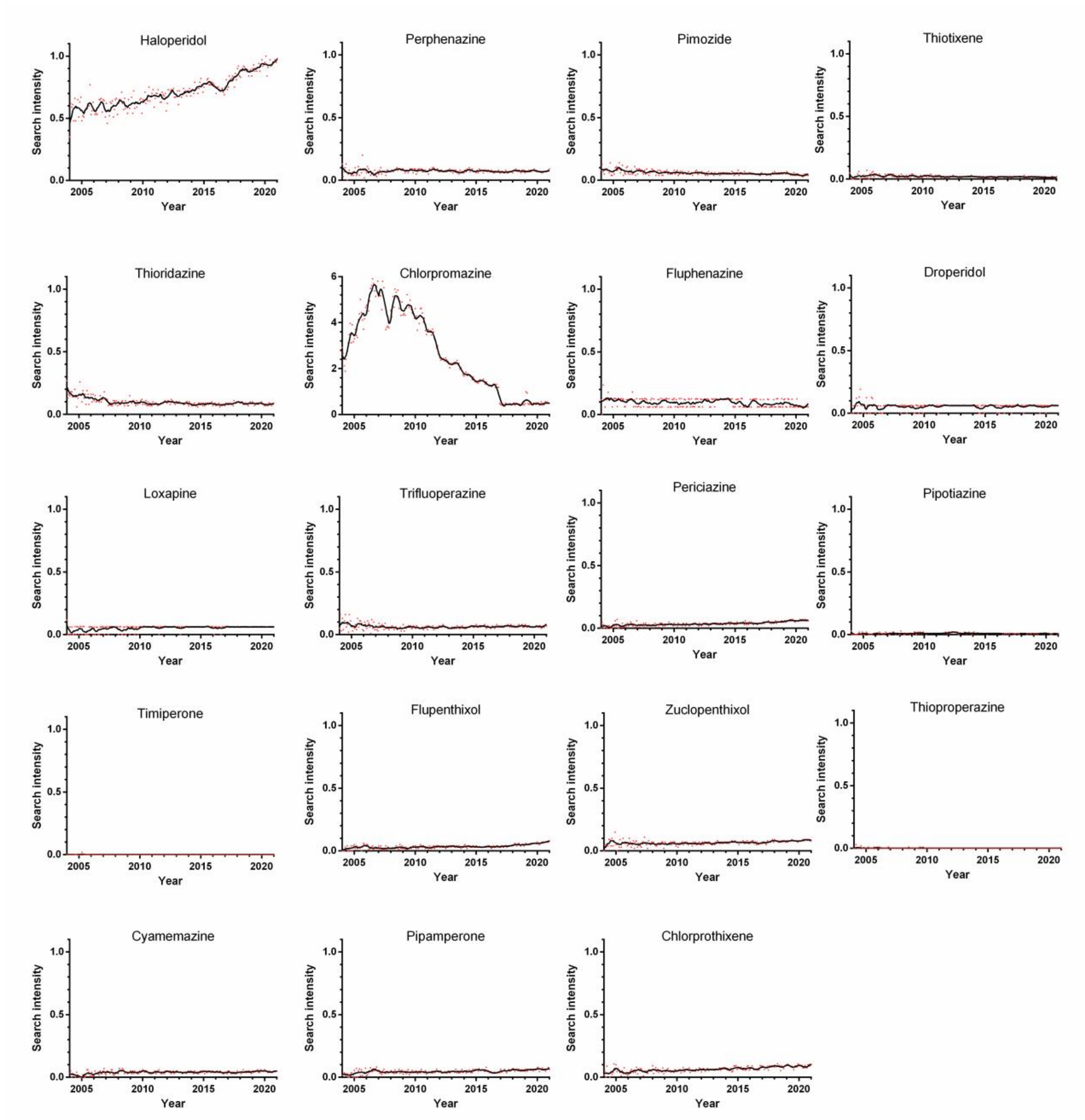

Supplementary Figure S5. Typical APD global search intensities in Jan 2004 – Jan 2021. 19 APDs are included and search intensities are normalized to the maximal intensity of haloperidol (see the first panel). Note the different y-axis used for chlorpromazine. Smooth polynomials of order 2 were adapted to 6 adjacent points (black).

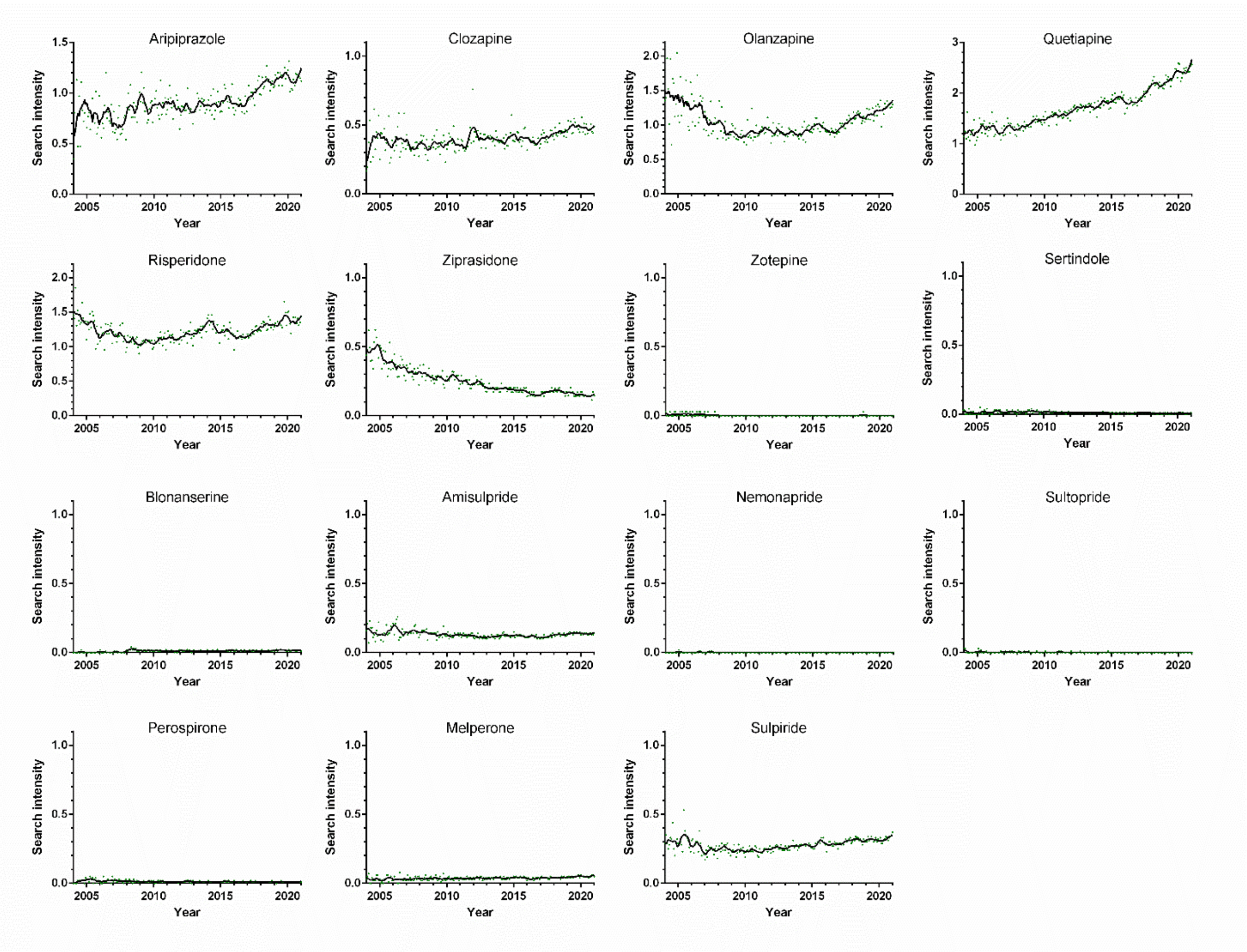

Supplementary Figure S6. Atypical APD global search intensities in Jan 2004 – Jan 2021. 15 APDs are included and search intensities are normalized to the maximal intensity of haloperidol (see Fig. S5). 7 additional (recently approved) atypical APDs are represented in Fig. 1 and Fig. S1. Note the different y-axes used for aripiprazole, olanzapine, quetiapine, and risperidone. Smooth polynomials of order 2 were adapted to 6 adjacent points (black).

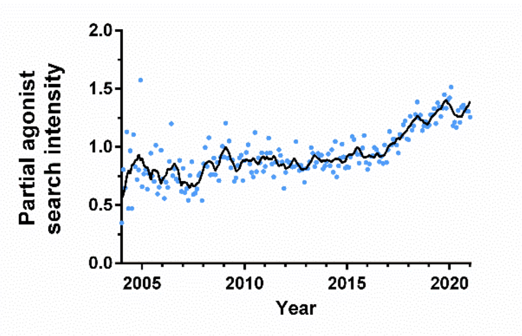

Supplementary Figure S7. Partial agonist search intensities in 2004-2020. The partial agonists include aripiprazole, brexpiprazole, and cariprazine. Lumateperone was not recognized as a partial agonist [2], and therefore excluded from the analysis. Search intensities were normalized to the maximum of haloperidol. Linear regression adapted to all data indicated a deviation from zero ($R^2 = 0.50$ and $p < 0.0001$). Smooth polynomials of order 2 were adapted to 6 adjacent points (black).

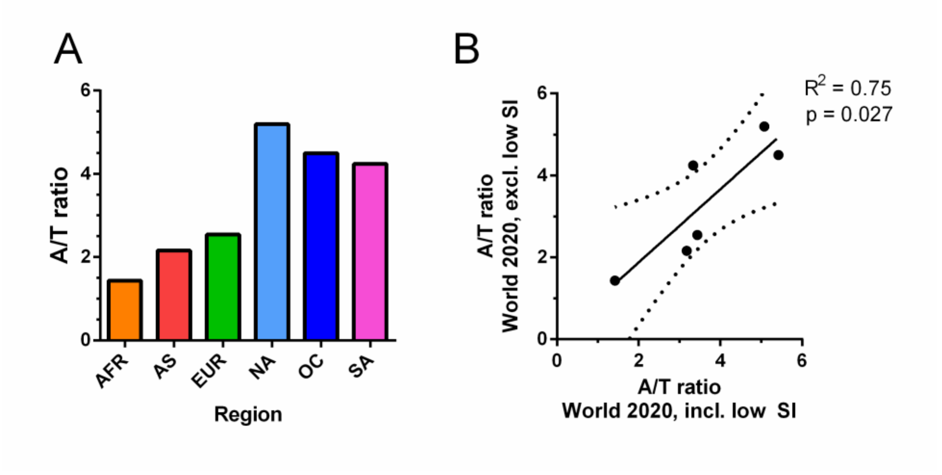

Supplementary Figure S8. Atypical/typical APD search intensity (A/T) ratios with low search intensity (SI) countries excluded. In total, 74 countries were included. A) Search intensity ratios per region. B) Correlation between A/T ratios for regions derived from data including and excluding low search intensity countries (linear regression, $R^2 = 0.75$ and $p = 0.027$; 95% C.I. is dotted). AFR, Africa (n = 8); AS, Asia (n = 21); EUR, Europe (n = 26); NA, North America (n = 8); OC, Oceania (n = 1); SA, South America (n = 10).

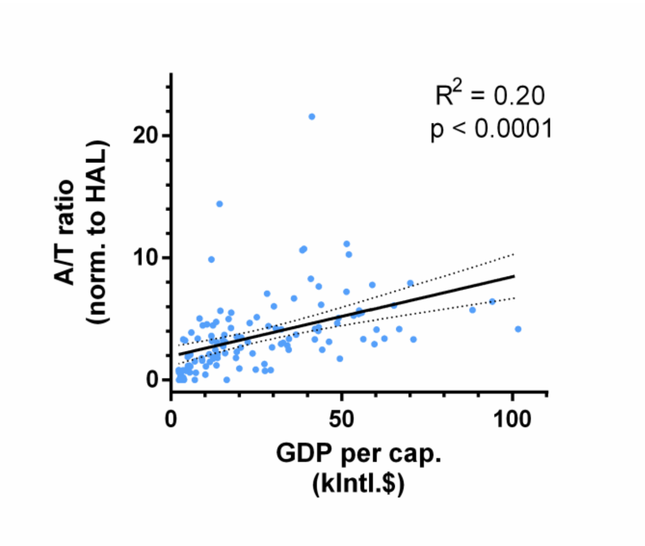

Supplementary Figure S9. Linear correlation of atypical/typical APD search intensity (A/T) ratios with GDP per capita (purchasing power parity). Data from 2020. A) Linear regression between global A/T ratios (normalized to haloperidol) and GDP per capita (R^2 = 0.20 and $p < 0.0001$; 95% C.I. is dotted). Low search intensity countries are included. GDP is expressed as thousand international dollars.

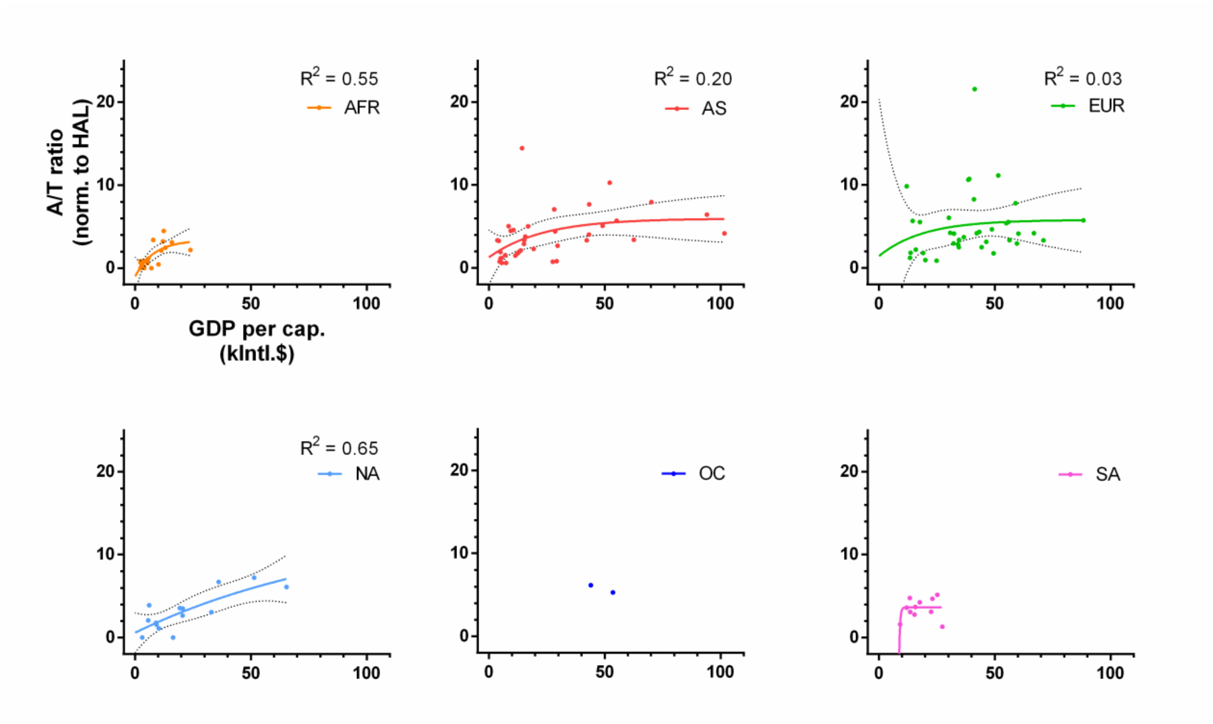

Supplementary Figure S10. Region-wise atypical/typical APD search intensity (A/T) ratios as functions of GDP per capita (purchasing power parity). Monoexponential functions were fitted to data points and 95% C.I.s are dotted. AFR, Africa (R^2 = 0.55, n = 21); AS, Asia (R^2 = 0.20, n = 37); EUR, Europe (R^2 = 0.03, n = 37); NA, North America (R^2 = 0.65, n = 14); OC, Oceania (N/D, n = 2), and SA, South America (ambiguous fit, n = 11).

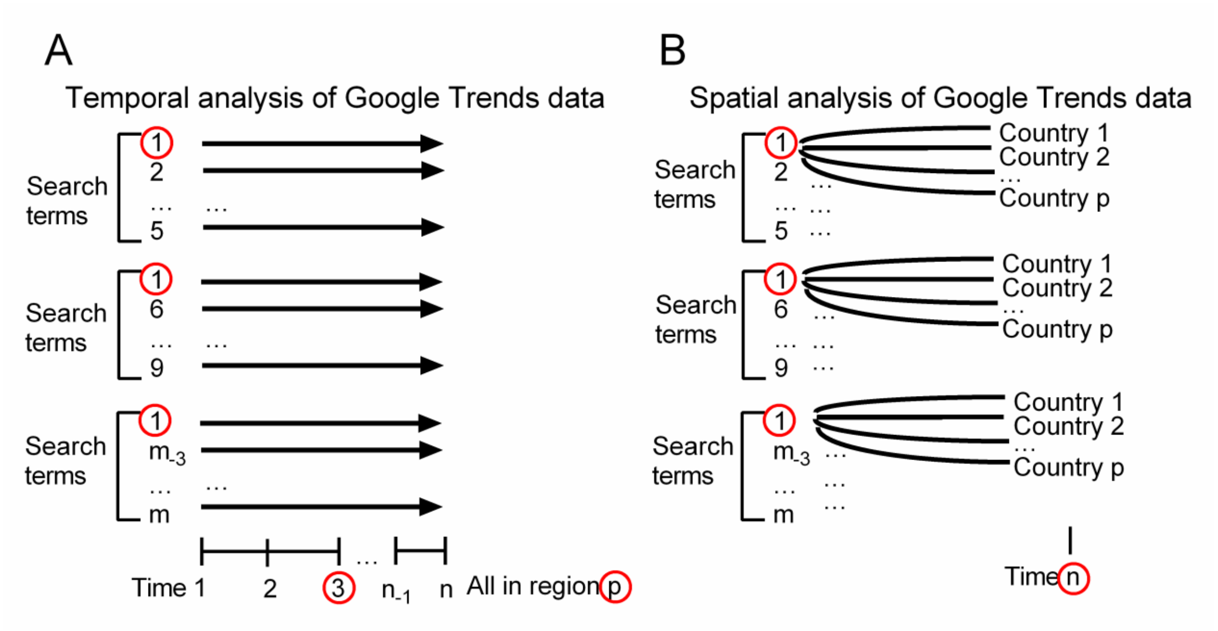

Supplementary Figure S11. Graphical representations of the data normalization principles. A) Temporal data were normalized to a specific drug (e.g., "haloperidol" or "risperidone") at a specific time point (e.g., maximum of "haloperidol" or "risperidone"). The selected region (e.g., "worldwide") was consistent for all searches. Inclusion of a specific drug and time point in all consecutive searches allowed for normalization of data. B) Spatial data were normalized to a specific drug in each country, at a specific time point (e.g., 2020). Inclusion of this drug, and use of the same time point and countries, in all consecutive searches allowed for normalization of data. The figure was rendered using GraphPad 6.

References

1 Bachmann, C. J. *et al.* International trends in clozapine use: a study in 17 countries. *Acta Psychiatr Scand* **136**, 37-51, doi:10.1111/acps.12742 (2017).

2 Zhang, L. & Hendrick, J. P. The presynaptic D2 partial agonist lumateperone acts as a postsynaptic D2 antagonist. *Matters* **4**, e201712000006 (2018).
